# Do games work? A meta-analytic synthesis of gamified learning for clinical reasoning in medical and allied health education

**DOI:** 10.1080/10872981.2026.2614233

**Published:** 2026-01-14

**Authors:** Ching-Yi Lee, Ching-Hsin Lee, Hung-Yi Lai, Po-Jui Chen, Mi-Mi Chen, Sze-Yuen Yau

**Affiliations:** a Department of Neurosurgery, Chang Gung Memorial Hospital at Linkou and Chang Gung University College of Medicine, Taoyuan, Taiwan; b Department of Radiation Oncology, Proton and Radiation Therapy Center, Chang Gung Memorial Hospital, Linkou, Taiwan; c (CG-MERC) Chang Gung Medical Education Research Centre, Linkou, Taiwan

**Keywords:** Gamification, clinical reasoning, health professions education, educational technology, meta-analysis

## Abstract

Gamification is increasingly adopted in health professions education to enhance clinical reasoning, a core competency essential for safe patient care. Although many interventions report positive outcomes, the magnitude and consistency of these effects remain uncertain. This meta-analytic review synthesizes quantitative findings on the effectiveness of gamified learning on clinical reasoning in medical and allied health learners across diverse contexts. Following PRISMA 2020 guidelines, we searched MEDLINE, Scopus, and Web of Science (2010–2023) for randomized and non-randomized studies evaluating gamified interventions targeting clinical reasoning. Eligible populations included pre- and post-licensure learners, with traditional or non-gamified instruction as comparators. Quantitative measures of clinical reasoning were required. Risk of bias was assessed using RoB 2.0 and ROBINS-I, and standardized mean differences (SMDs) were pooled using a random-effects model. From 713 records, 26 studies met inclusion criteria and 10 contributed to the meta-analysis. Gamified interventions were associated with improved clinical reasoning compared with traditional instruction (SMD = 1.11; 95% CI: 0.69–1.52). Substantial heterogeneity was observed (I² = 85%). Assessment of publication bias suggested possible overestimation of effects, with an adjusted pooled estimate of 0.75 (95% CI: 0.24–1.27). The certainty of evidence was rated as low due to heterogeneity, risk of bias, and potential publication bias. Gamified learning may support the development of clinical reasoning in health professions education; however, considerable variability across studies and low certainty of evidence warrant cautious interpretation. Future research should employ theory-informed designs, validated reasoning measures, and rigorous methodologies to clarify when and how gamification is most effective.

## Introduction

Clinical reasoning—the iterative process of gathering cues, generating and refining hypotheses, and selecting appropriate management options—is widely recognised as a core competency of health professionals [1]. Strong reasoning skills are linked to reduced diagnostic errors, improved therapeutic decisions, and ultimately, better patient outcomes [2]. However, clinical reasoning is notoriously difficult to teach. Traditional approaches such as didactic instruction and static case discussions often lack opportunities for feedback, adaptive challenge, and practice under uncertainty [3]. Consequently, there is growing advocacy for active, feedback-rich, and engaging learning environments that support higher-order cognition [4].

In response to these instructional challenges, gamification—the application of game design elements (e.g., rules, points, narrative, immediate feedback) in non-game contexts—has emerged as a promising pedagogical approach [5]. Gamified learning environments leverage motivational and cognitive mechanisms to enhance intrinsic motivation (self-determination theory) [6], support engagement and challenge–skill balance (flow theory) [7], and optimise cognitive processing to promote schema construction (cognitive load theory) [8]. Gamified approaches include both digital and non-digital strategies, ranging from serious games, escape rooms, branching case scenarios, tabletop games, and digital quizzes, offering safe and repeatable opportunities to practise decision-making in dynamic clinical contexts without patient risk [9]. In this review, we refer to gamification modalities as both specific mechanics (e.g., points, leaderboards, badges, narrative, adaptive feedback) and broader instructional formats (e.g., branching case scenarios, tabletop simulations, and scenario-based role-play tasks). Different game mechanics may align with specific reasoning subprocesses, such as hypothesis generation, diagnostic trade-offs, and metacognitive monitoring.

### Why focus on clinical reasoning?

Despite the growing use of gamification in health professions education, recent reviews indicate that serious games and gamified tools can enhance knowledge and procedural skills [10,11], while clinical reasoning is less frequently evaluated as a primary outcome. Clinical reasoning encompasses cue acquisition, hypothesis generation, diagnostic formulation, and metacognitive monitoring [12], yet many gamified interventions focus on knowledge recall or skill performance rather than reasoning processes. Moreover, prior syntheses often aggregate diverse gamified approaches without examining how specific design elements influence reasoning development [9]. A more granular understanding of how particular mechanics align with reasoning subprocesses is therefore required. Although emerging studies across health professions education suggest that gamified approaches may support clinical reasoning across a range of digital and non-digital formats—including case-based games, tabletop simulations, and collaborative platforms [13–18] —the evidence remains methodologically and conceptually heterogeneous.

### Residual challenges

Notwithstanding these promising developments, the literature remains fragmented. Study designs range from uncontrolled pre–post surveys to randomised trials, outcome measures vary widely, and theoretical rationales are inconsistently articulated. Furthermore, most studies are small, single-centre endeavours, limiting generalisability, and few examine sustained behavioural change or patient-level effects. Many interventions also employ researcher-developed or non-validated reasoning assessments, limiting comparability across settings. Importantly, alignment between specific game mechanics and reasoning subprocesses is rarely examined, leaving the mechanisms by which gamification supports reasoning insufficiently understood.

These limitations are consistent with findings from our prior scoping review, which identified 53 gamified interventions targeting clinical reasoning across diverse health professions programs [19]. While mapping contexts, mechanics, and theories, it highlighted persistent gaps: limited use of validated reasoning assessments, underutilisation of cognitive frameworks, methodological heterogeneity, and minimal longitudinal evaluation. Accordingly, a quantitative synthesis is needed to estimate effect magnitude and explore the relationship between game design and reasoning outcomes. Explicit consideration of how learning theories align with reasoning demands may further enhance conceptual clarity and interpretation of findings.

### Purpose of the present review

In response to these identified gaps, we conducted a systematic review and meta-analysis focused explicitly on gamified interventions designed to enhance clinical reasoning in medical and allied health education. Our objectives are to: (i) synthesise the quantitative evidence for effectiveness on validated reasoning outcomes; (ii) produce a narrative typology of game formats and mechanics; (iii) appraise methodological and theoretical quality; and (iv) explore moderators such as learner level, profession, delivery modality, and alignment with instructional design principles. By linking design features to cognitive outcomes, the review aims to provide actionable guidance for curriculum developers and identify priorities for future research. In doing so, it addresses critical methodological and conceptual gaps in prior literature and clarifies the pedagogical mechanisms through which gamification may support clinical reasoning.

## Materials and methods

This systematic review and meta-analysis represent an elaborative extension of our previously published scoping review on gamification in clinical reasoning education [19]. As this review evolved from a previously completed scoping review, it was not prospectively registered in PROSPERO or another public registry; we acknowledge this as a limitation with respect to transparency. To mitigate this, the review was conducted in accordance with Systematic Reviews and Meta-Analyses (PRISMA) 2020 recommendations [20], with eligibility criteria, outcomes, and analytic methods specified a priori, along with independent screening, structured data extraction, and formal risk of bias assessment to ensure methodological rigour.

### Eligibility criteria

Eligibility criteria were developed using the PICOTS framework (Population, Intervention, Comparator, Outcome, Timing, Setting), as outlined below:

- **Population:** Pre- and post-licensure learners in health professions education, including but not limited to medical, nursing, pharmacy, and allied health students.
- **Intervention:** Any gamified educational intervention explicitly aimed at developing clinical reasoning skills. This included digital serious games, escape rooms, branching scenario simulations, and immersive gamified simulations.
- **Comparator:** Traditional or non-gamified instructional strategies (e.g., lectures, tutorials, non-interactive case studies), no intervention, or pre-post comparison within the same group.
- **Outcomes:** Quantitative measures of clinical reasoning competence or performance, such as diagnostic accuracy, problem-solving ability, script concordance scores, decision-making under uncertainty, or performance in OSCE stations with reasoning criteria.
- **Study designs:** Randomised controlled trials (RCTs), quasi-experimental studies, controlled before-after studies, and high-quality observational studies with valid outcome measures.
- **Setting:** Any formal educational context (undergraduate, postgraduate, or continuing education) within healthcare.

Qualitative-only studies, opinion papers, protocols without results, and studies focusing solely on knowledge acquisition or psychomotor skills without a reasoning component were excluded.

### Information sources and search strategy

A comprehensive search strategy was developed in consultation with a health sciences librarian. The following electronic databases were searched from January 2010 to December 2023: MEDLINE (via PubMed), Scopus, and Web of Science. Search terms combined controlled vocabulary (e.g., MeSH) and free-text keywords related to “*gamification*,” “*serious games*,” “*clinical reasoning*,” and “*health professions education*.” Boolean operators, truncation, and proximity searching were used to optimise sensitivity. Reference lists of included studies and relevant reviews were hand-searched to identify additional eligible literature. The same search strategy as in our prior scoping review was applied [19].

### Study selection

All search results were imported into EndNote (version 20) for de-duplication, and then uploaded into Covidence for screening. Two reviewers independently screened all titles/abstracts and full texts. Discrepancies were resolved through discussion or consultation with a third reviewer. The screening process is summarised in a PRISMA 2020 flow diagram.

### Data extraction

A structured data extraction form was developed and pilot-tested on a subset of included studies. Extracted data included: (1) bibliographic details; (2) population characteristics (discipline, learner level, sample size); (3) gamification characteristics (type, delivery mode, duration, design elements); (4) theoretical framework (if reported); (5) comparator intervention; (6) clinical reasoning outcome(s) measured; (7) assessment tools and timing; (8) key findings (effect sizes, significance); and (9) risk of bias indicators. Data were extracted independently by two reviewers, with discrepancies resolved through consensus.

### Risk of bias assessment

Risk of bias was assessed independently by two reviewers using appropriate tools. The Cochrane Risk of Bias 2.0 (RoB 2) tool [21] was used for RCTs, and the ROBINS-I tool [22] for non-randomised studies. Discrepancies were resolved through discussion.

### Data synthesis

We conducted both narrative and quantitative syntheses, depending on data availability and comparability across studies. All included studies were first synthesised narratively, structured by the type of gamified intervention, learner population, theoretical framework, and type of clinical reasoning outcome.

For studies that reported sufficient quantitative data, a meta-analysis was performed using R software (version 4.3.1) with the “meta” and “metafor” packages. Continuous outcomes were pooled using standardised mean differences (SMDs) with 95% confidence intervals, employing a random-effects model (DerSimonian and Laird method) to account for heterogeneity across educational settings and outcome tools.

Effect sizes were calculated primarily from post-test scores, with gain scores examined in sensitivity analyses when available. Statistical heterogeneity was assessed using I², τ², and the Q test, and a 95% prediction interval was calculated [23].

## Results

**Figure 1.**
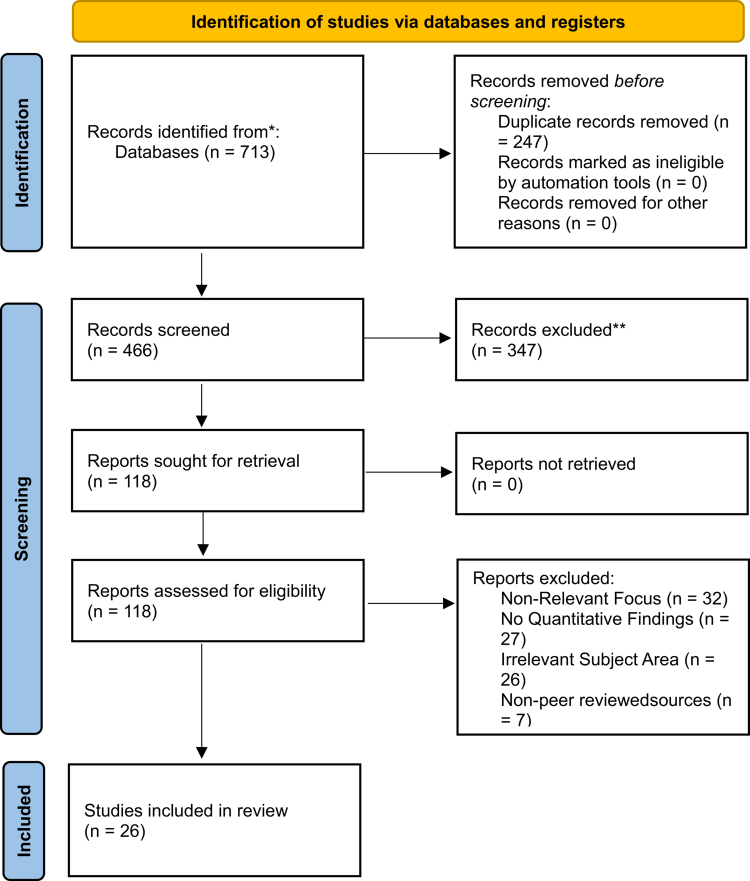
PRISMA 2020 Flow Diagram of Study Selection for Gamified Clinical Reasoning Interventions in Health Professions Education. Note: This diagram illustrates the identification, screening, eligibility assessment, and inclusion process for studies examining gamified interventions targeting clinical reasoning in health professions education. A total of 713 records were identified through database searching, with 466 records screened after removing duplicates. Following full-text review of 118 articles, 26 studies met inclusion criteria. Common reasons for exclusion included absence of clinical reasoning outcomes, non-gamified instructional formats, and insufficient quantitative data reporting. This flow diagram reflects a rigorous, transparent selection process consistent with PRISMA 2020 guidance.

A systematic search across electronic databases yielded 713 records. After removal of duplicates, 466 unique records remained for title and abstract screening. Of these, 347 were excluded for irrelevance or insufficient data. A total of 118 full-text articles were assessed for eligibility, with 65 subsequently excluded based on predefined criteria (e.g., non-peer-reviewed, off-topic, or exclusively qualitative design). This process resulted in 26 eligible studies published between 2014 and 2023 (Figure 1).

### Study design and participant characteristics

As summarised in Table 1, publication volume increased over time, with over one-third of included studies published in 2022 (34.6%, *n* = 9). Studies were conducted across multiple countries, most commonly in Canada and the United States (both 15.4%, *n* = 4), and were predominantly situated in university-based (34.6%, *n* = 9) or medical school settings (23.1%, *n* = 6). Randomised controlled trials (26.9%, *n* = 7) and quasi-experimental designs (26.9%, *n* = 7) were most frequent, together accounting for over half of the included studies. Participants were primarily medical (42.3%, *n* = 11) and nursing learners (34.6%, *n* = 9), with a strong emphasis on undergraduate education (69.2%, *n* = 18). Fewer studies involved postgraduate trainees or interprofessional cohorts.

**Table 1.** Characteristics of included studies evaluating gamified learning interventions for clinical reasoning (*N* = 26).

Study Characteristics		*n* = 26	%
Year	2014	1	3.85
	2015	1	3.85
	2016	1	3.85
	2017	3	11.54
	2018	2	7.69
	2019	1	3.85
	2020	1	3.85
	2021	4	15.38
	2022	9	34.62
	2023	3	11.54
Country	Canada	4	15.38
	USA	4	15.38
	Germany	3	11.54
	India	2	7.69
	South Korea	2	7.69
	Other (Canada, Cyprus, Egypt, France, Hong Kong, Italy, Morocco, Netherlands, Norway, Philippines, UK)	11	42.3
Language	English	26	100
Research design	Quasi-Experimental Design	7	26.92
	Randomised Controlled Trial	7	26.92
	Pre-Post Design (Non-Comparative)	5	19.23
	Development/Usability Study	2	7.69
	Mixed-Methods/Descriptive Study	2	7.69
	Non-Empirical/Descriptive	2	7.69
	Survey-Based Study	1	3.85
Study Setting	University-Based Health Professions Programs	9	34.62
	Medical School Settings	6	23.08
	Clinical Simulation Centres	3	11.54
	Residency and Postgraduate Programs	3	11.54
	Departmental/Faculty-Based Settings	2	7.69
	Hospital-Based Clinical Units	2	7.69
	Dental and Interdisciplinary Institutions	1	3.85
Specialty	Medicine	11	42.3
	Nursing	9	34.6
	Physical Medicine and Rehabilitation	3	11.5
	Other (Dentistry, Midwifery, Paediatrics and Interprofessional Teams, Psychiatry)	3	11.5
Education level	Undergraduate	18	69.23
	Mixed Level (Undergrad + Postgrad)	7	26.92
	Professional (Licensed Practitioners)	1	3.85

Note: This table summarises the key characteristics of the 26 included studies, demonstrating a rapidly expanding evidence base for gamification in clinical reasoning education. Most studies were conducted in undergraduate medical or nursing programs, reflecting a primary focus on pre-licensure learners. Digital serious games, gamified e-learning modules, and simulation-based exercises were the most frequently reported formats, although non-digital approaches (e.g., card-based simulations, escape rooms) also appeared. Most interventions were short-duration and delivered in single-centre academic settings, with few long-term or multi-institution trials. While this reflects early adoption and feasibility-focused research in the field, it also underscores the need for larger-scale and longitudinal evaluations.

### Game characteristics and theoretical grounding

Table 2 summarises the characteristics of gamified interventions and their theoretical foundations. Digital formats—particularly serious games (30.8%, *n* = 8) and gamified e-learning modules (23.1%, *n* = 6)—were most common, although a substantial minority employed physical tabletop games or simulation-based activities. Across formats, interventions most frequently emphasised scenario-based decision making (69.2%, *n* = 18), structured feedback (61.5%, *n* = 16), and progress tracking (65.4%, *n* = 17), reflecting a focus on iterative clinical reasoning practice rather than isolated knowledge acquisition. Competitive and immersive elements were present in approximately one-quarter of studies (*n* = 7).

**Table 2.** Gamification elements, learning theories, and delivery modalities across included studies.

Game design characteristics		*n* = 26	%
Game type	Digital Serious Games	8	30.77
	Gamified e-Learning Modules	6	23.08
	Simulation-based Learning	4	15.38
	Physical Board Games	4	15.38
	Digital Simulations	3	11.54
	Other/Not Specified	1	3.85
Platform	Web-based/Online	8	30.77
	Computer-based	7	26.92
	Simulation/In-person	5	19.23
	Physical Tabletop	4	15.38
	Mobile-based	2	7.69
Game Mechanics	Scenario-Based Decision Making	18	69.23
	Scoring and Progress Tracking	17	65.38
	Feedback and Reflection	16	61.54
	Competition and Leaderboards	7	26.92
	Simulation and Immersion	7	26.92
	Gamified Rewards and Levels	2	7.69
Theoretical Framework	Clinical Reasoning Frameworks	10	38.46
	Experiential and Situated Learning Theories	9	34.62
	Not Explicitly Theorised	3	11.54
	Cognitive and Dual Process Theories	2	7.69
	Ethical and Relational Theories	1	3.85
	Motivation and Self-Regulation Theories	1	3.85

Note: Table 2 highlights the distribution of game mechanics and theoretical approaches across interventions. Scenario-based decision-making, immediate feedback, and reward or point systems were the most employed mechanics, suggesting an emphasis on scaffolding diagnostic decisions and reinforcing performance. Narrative, time pressure, and collaboration features were less frequently used, despite relevance for simulating authentic clinical contexts. Theoretical grounding varied considerably: some studies referenced cognitive load theory, experiential learning, self-determination theory, or dual-process reasoning frameworks, while others lacked explicit conceptual justification. This heterogeneity reflects the field’s developmental stage and suggests opportunities to enhance deliberate theory-to-design alignment in future interventions.

Theoretical grounding was heterogeneous. While some studies explicitly drew on clinical reasoning frameworks (38.5%, *n* = 10) or experiential and situated learning theories (34.6%, *n* = 9), 11.5% of studies (*n* = 3) reported no explicit theoretical framework, indicating substantial variability in the depth and specificity of conceptual integration underpinning gamified clinical reasoning interventions.

### Meta-analysis of effectiveness

Ten of the 26 included studies provided comparable quantitative data for meta-analysis (*N* = 931 learners; 467 interventions, 464 control), with sample sizes ranging from 35 to 168 participants. As summarised in Table 3, most studies assessed clinical reasoning directly using performance-based or standardised reasoning measures, while a minority employed validated self-report instruments or reasoning-relevant knowledge tests.

**Table 3.** Effect size estimates for gamified interventions compared with control or pre-test conditions.

Study	Experimental	Control	SMD	95% CI	Weight	*t*	*p*-value
Badr, 2022	40 (20.8/1.8)	40 (17.1/2.1)	1.874	1.344–2.403	9.82		
Chon et al., 2019	37 (58.43/4.12)	35 (51.34/4.77)	1.577	1.044–2.109	9.81		
Kim et al., 2022	38 (59.55/3.9)	38 (52.92/4.42)	1.574	1.057–2.092	9.89		
Maheu-Cadotte et al., 2023	60 (70.8/8.7)	60 (64/9.1)	0.759	0.388–1.13	10.69		
Nicolaidou et al., 2015	17 (14.12/1.69)	18 (10.11/1.51)	2.449	1.55–3.348	7.56		
Novoseltseva et al., 2022	28 (60.5/7.1)	27 (50.1/7.5)	1.405	0.81–1.999	9.43		
Raupach et al., 2021	81 (15.6/3.4)	87 (14.1/3.5)	0.433	0.126–0.739	10.98		
Schwarzkopf et al., 2023	60 (84.3/8.1)	55 (79.5/9.3)	0.548	0.176–0.921	10.68		
Wong et al., 2022	64 (28.1/3.2)	61 (25.6/3.8)	0.709	0.347–1.071	10.73		
Zairi et al., 2022	42 (15.24/2.5)	43 (14.48/2.8)	0.284	−0.855	10.4		
Random effects model	42.744/4.451	37.925/4.88	1.105	0.688–1.521	99.99	5	<.001

	Parameter	Value	95% CI
Quantifying heterogeneity			
1	*τ* ^2^	0.39	0.151–1.604
2	*τ*	0.62	0.388–1.266
3	I^2^	0.85	0.75–0.915
4	H	2.62	1.998–3.429

	Q	d.f.	*p*-value
Test of heterogeneity	61.67	9	<.001

Note: Effect sizes (SMDs) varied across studies but generally favoured gamified learning, with several reporting moderate to large improvements in clinical reasoning outcomes. This pattern supports the potential utility of gamification in fostering higher-order decision-making skills. However, variability in effect magnitudes likely reflects differences in learner background, instructional scaffolding, fidelity of game design, and the type and validity of reasoning outcome measures used. Studies employing structured or validated assessment tools tended to show more moderate and consistent effects than those using researcher-developed instruments, highlighting the importance of robust outcome measurement. These findings align with the broader narrative of a promising yet methodologically diverse and emerging research domain.

#### Pooled effects

**Figure 2.**
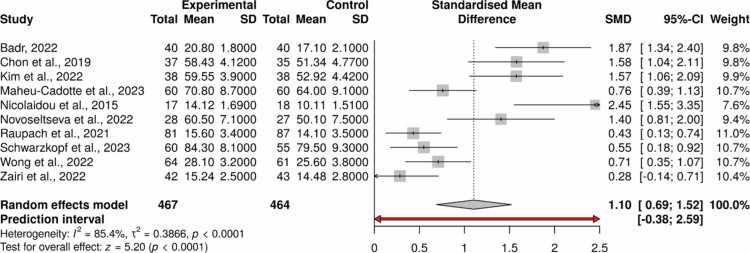
Forest Plot of Standardised Mean Differences Comparing Gamified Interventions to Control or Pre-Test Conditions on Clinical Reasoning Outcomes (Random-Effects Model) Note: The forest plot demonstrates the standardised mean differences (SMDs) for studies assessing the impact of gamified learning on clinical reasoning performance. The pooled random-effects estimate indicates a moderate-to-large positive effect in favour of gamified interventions. Individual effects vary in magnitude, reflecting diversity in learner groups, game mechanics, outcome measures, and implementation settings. The I² value of 86% indicates substantial heterogeneity, suggesting that effects differ meaningfully across educational contexts. Error bars represent 95% confidence intervals; studies are weighted by inverse variance.

Gamified interventions were associated with a large and statistically significant improvement in clinical reasoning (SMD = 1.11, 95% CI: 0.69 to 1.52, *p* < 0.0001; Figure 2). Substantial heterogeneity was observed (*I²* = 85.4%; *τ²* = 0.39), with a wide prediction interval (–0.38 to 2.59), indicating considerable variability in effects across contexts. Subgroup analyses were not conducted due to the limited number of studies with comparable outcome data.

#### Risk of bias assessment

Risk of bias was assessed using RoB 2.0 for randomised trials and ROBINS-I for non-randomised studies (Supplementary Table 1). Overall, four studies were judged to have low risk of bias, four had moderate risk, and two raised some concerns. Sensitivity analysis excluding the latter did not materially change the pooled effect (SMD = 1.09, 95% CI: 0.69 to 1.48), supporting the robustness of the findings.

#### Publication bias

**Figure 3.**
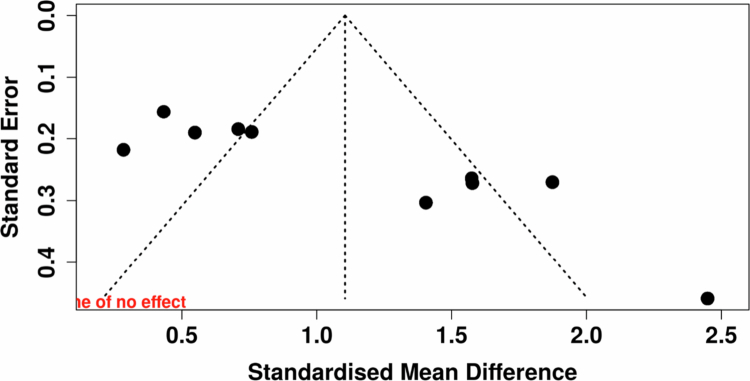
Funnel Plot for Publication Bias Assessment Among Studies Included in the Meta-Analysis. Note: The funnel plot visualises the distribution of effect sizes and standard errors for included studies. Mild asymmetry is observed, with a slight clustering of smaller studies reporting larger positive effects. This pattern suggests potential publication bias and underscores the need for cautious interpretation of pooled effects. Visual inspection supports quantitative tests indicating possible bias favoring positive findings in this emerging field. Vertical line indicates pooled effect estimate; diagonal lines represent pseudo-95% confidence limits.

**Figure 4.**
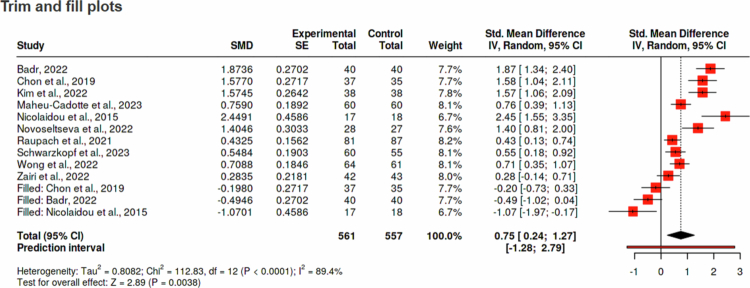
Trim-and-fill adjusted forest plot of gamified interventions on clinical reasoning outcomes. Note: This figure presents the trim-and-fill analysis used to assess the potential influence of publication bias on the estimated effect of gamified learning on clinical reasoning. Each square represents the standardised mean difference (SMD) for a study, with square size proportional to the study weight. Horizontal bars indicate 95% confidence intervals. The filled red squares labelled as “Filled” represent statistically imputed studies estimated to be missing due to publication bias. The diamond at the bottom displays the overall pooled effect estimate under the random-effects model after the trim-and-fill adjustment.

Funnel plot asymmetry suggested potential publication bias (Figure 3). Trim-and-fill analysis imputed three missing studies, yielding an adjusted pooled effect of 0.75 (95% CI: 0.24 to 1.27; Figure 4), which remained statistically significant but indicated possible overestimation of the observed effect.

#### GRADE assessment

Using the GRADE framework, the certainty of evidence for the pooled effect was rated as low. Downgrading reflected the cumulative impact of study-level risk of bias, substantial between-study heterogeneity, and potential publication bias. No serious concerns were identified regarding indirectness or imprecision of the pooled estimate.

## Discussion

This systematic review and meta-analysis provide quantitative evidence for the effectiveness of gamified educational interventions in enhancing clinical reasoning among learners in health professions education. The pooled standardised mean difference (SMD = 1.11) indicates a large improvement relative to traditional or non-gamified instruction, extending prior scoping reviews [19] by offering meta-analytic confirmation of effectiveness. To our knowledge, this is the first synthesis to demonstrate that integrating game mechanics—such as scenario-based decision making, feedback loops, and progress tracking—can yield substantial gains in applied clinical reasoning performance.

Across included studies, gamified approaches appeared particularly effective for promoting applied reasoning, within simulated or case-based contexts, frequently targeting diagnostic accuracy and decision making under uncertainty. These findings are consistent with cognitive perspectives on clinical reasoning, which emphasise the development and refinement of illness scripts, forward reasoning, and hypothesis testing through repeated exposure to authentic problems [3,24]. Features commonly embedded in gamified interventions—such as immediate feedback, adaptive challenge, and iterative decision cycles—align with principles of deliberate practice and self-regulated learning, supporting metacognitive monitoring and progressive skill refinement [25,26].

### Theoretical and pedagogical implications

Many interventions were explicitly informed by clinical reasoning frameworks [27], script theory [28], or experiential learning models [29], providing conceptual grounding for decision-rich learning environments. However, a notable proportion of studies lacked an articulated theoretical framework, echoing limitations identified in earlier reviews [30,31]. This heterogeneity in theoretical grounding reflects the relatively early developmental stage of gamified clinical reasoning pedagogy and constrains theory-driven comparisons of effectiveness. Future research with more consistent theoretical reporting may allow examination of whether explicit alignment with cognitive, motivational, or reasoning theories moderates learning outcomes.

Digital serious games and gamified e-learning modules were the most commonly used formats, highlighting their scalability and accessibility. Nevertheless, analogue approaches—such as tabletop games and in-person simulations—also demonstrated positive effects, indicating that digital delivery is not a prerequisite for cognitive impact. Across formats, interventions that incorporated meaningful learner choice, adaptive challenge, and informative feedback appeared better aligned with established motivational and engagement principles than designs relying primarily on competition [6,7]. From a pedagogical perspective, effectiveness appears to depend less on delivery medium than on alignment between game mechanics and cognitive–motivational processes relevant to clinical reasoning.

### Heterogeneity and contextual variability

Substantial heterogeneity was observed across studies, suggesting that variation in effects reflects genuine differences in intervention design, learner populations, delivery formats, and outcome measurement rather than random error. Such variability is common in complex educational interventions and may be pedagogically informative [32]. The wide prediction interval indicates that while many gamified interventions are likely to be beneficial, others may yield limited or negligible effects, underscoring the importance of context-sensitive design and instructional alignment.

Only a subset of eligible studies contributed to the meta-analysis, limiting synthesis power and representativeness. Several studies relied on small samples or researcher-developed outcome measures, reducing confidence in the precision and generalisability of the pooled estimate. These considerations are reflected in the low certainty rating assigned using the GRADE framework, driven by cumulative study-level risk of bias, inconsistency, and potential publication bias. Accordingly, the pooled effect should be interpreted as an average estimate within methodologically diverse and evolving evidence base rather than as evidence of uniform benefit.

Learning outcomes may also be moderated by contextual and learner-related factors, including baseline motivation, prior gaming experience, digital literacy, and institutional readiness for technology-enhanced learning [33]. Collaborative and team-based gamification formats may further leverage social learning mechanisms, whereby peer interaction and shared problem solving enhance reasoning processes [34]. Conversely, poorly scaffolded or overly complex designs may impose extraneous cognitive load and attenuate learning gains. These considerations highlight the need to align gamified interventions with local resources, learner characteristics, and pedagogical objectives.

### Methodological considerations and implications for future research

The methodological characteristics of the included studies have important implications for interpreting the observed effects. While sensitivity analyses suggested that study-level risks of bias did not materially alter the pooled estimate, the presence of substantial heterogeneity and potential publication bias indicates that effect sizes should be interpreted with caution. These features are common in complex educational interventions and highlight the importance of contextual variability when translating findings into practice.

Compared with meta-analyses of simulation-based learning [35], which often report more modest effects, the present findings suggest that engagement-enhancing features of gamification may amplify learning beyond observational or passive simulation alone. However, given variability in study design, implementation, and outcome measurement, gamification should be regarded as a promising but still emergent pedagogical strategy. Continued theory-informed design, rigorous evaluation, and cumulative evidence synthesis will be essential to clarify when, how, and for whom gamified learning most effectively supports clinical reasoning development.

### Limitations and future research

#### Methodological limitations

First, this review was not prospectively registered, as it was conducted as an extension of a previously published scoping review. Although PRISMA 2020 guidance was followed, the absence of prospective registration may limit transparency regarding a priori analytic decisions. Substantial heterogeneity was observed across studies, likely reflecting variation in intervention design, duration, implementation fidelity, and outcome measurement. In addition, common risks of bias—including lack of blinding and incomplete allocation concealment—may have influenced effect estimates. Evidence of potential publication bias further suggests that pooled effects may be overestimated, although the limited number of studies constrains the robustness of such assessments.

#### Conceptual and measurement limitations

Interventions varied widely in their theoretical grounding, and some lacked explicit alignment with established learning or clinical reasoning frameworks, limiting theory-driven interpretation. Several studies also relied on researcher-developed outcome measures, which may reduce comparability and precision despite reported content validation.

#### Reporting and evidence limitations

Only a subset of eligible studies contributed to the meta-analysis, limiting statistical power and representativeness. The exclusive focus on quantitative outcomes also meant that qualitative insights into learner experience, design processes, and contextual implementation were not synthesised.

#### Implications for future research

Future research should prioritise rigorously designed and transparently reported studies, including prospective registration, predefined analysis plans, and adequately powered randomised trials. Interventions should be explicitly grounded in established theories of clinical reasoning and learning to ensure alignment between game mechanics and underlying cognitive processes. Validated, theory-based outcome measures are needed to improve precision and comparability. Longitudinal and mixed-methods studies are needed to examine retention, transfer, learner experience, and contextual influences. Comparative studies across gamification modalities may clarify which designs are most effective for specific learner groups and educational contexts. As the evidence base matures, moderator analyses and meta-regression will be essential to determine under what conditions gamification most effectively supports clinical reasoning development.

## Conclusion

This systematic review and meta-analysis suggest that gamified learning interventions may support the development of clinical reasoning among learners in medical and allied health education. Across diverse educational contexts, gamification was associated with improvements in applied reasoning outcomes, including diagnostic thinking and decision making under uncertainty. However, the certainty of evidence was low and substantial variability across studies was observed, indicating that these findings should be interpreted cautiously. Rather than demonstrating uniform effectiveness, the current evidence highlights the potential of well-designed gamified approaches to complement existing instructional strategies when aligned with learning objectives and contextual needs. Gamification should therefore be regarded as promising but still emerging pedagogical approach, warranting careful implementation and ongoing evaluation. Further theory-informed and methodologically rigorous research is required to clarify when, how, and for whom gamified learning most effectively supports clinical reasoning development.

## Supplementary Material

PRISMA_2020_checklistV1.docx PRISMA_2020_checklistV1.docx

## Data Availability

All data supporting the findings of this review are derived from published studies and are fully cited within the manuscript. No new datasets were generated or analysed beyond those reported in the included literature.
